# Nonintegrating Direct Conversion Using mRNA into Hepatocyte-Like Cells

**DOI:** 10.1155/2018/8240567

**Published:** 2018-09-20

**Authors:** Sangtae Yoon, Kyojin Kang, Young-duck Cho, Yohan Kim, Elina Maria Buisson, Ji-Hye Yim, Seung Bum Lee, Ki-Young Ryu, Jaemin Jeong, Dongho Choi

**Affiliations:** ^1^HY Indang Center of Regenerative Medicine and Stem Cell Research, Hanyang University, Seoul 04763, Republic of Korea; ^2^Department of Surgery, Hanyang University College of Medicine, Seoul 04763, Republic of Korea; ^3^Department of Emergency Medicine, Korea University Guro Hospital, Seoul 02841, Republic of Korea; ^4^Laboratory of Radiation Exposure & Therapeutics, National Radiation Emergency Medical Center, Korea Institute of Radiological & Medical Science (KIRAMS), Seoul 01812, Republic of Korea; ^5^Department of Obstetrics and Gynecology, Hanyang University College of Medicine, Seoul 04763, Republic of Korea

## Abstract

Recently, several researchers have reported that direct reprogramming techniques can be used to differentiate fibroblasts into hepatocyte-like cells without a pluripotent intermediate step. However, the use of viral vectors for conversion continues to pose important challenges in terms of genome integration. Herein, we propose a new method of direct conversion without genome integration with potential clinical applications. To generate hepatocyte-like cells, mRNA coding for the hepatic transcription factors Foxa3 and HNF4*α* was transfected into mouse embryonic fibroblasts. After 10-12 days, the fibroblasts converted to an epithelial morphology and generated colonies of hepatocyte-like cells (R-iHeps). The generated R-iHeps expressed hepatocyte-specific marker genes and proteins, including albumin, alpha-fetoprotein, HNF4*α*, CK18, and CYP1A2. To evaluate hepatic function, indocyanine green uptake, periodic acid-Schiff staining, and albumin secretion were assessed. Furthermore, mCherry-positive R-iHeps were engrafted in the liver of Alb-TRECK/SCID mice, and we confirmed FAH enzyme expression in Fah^1R^Tyr^c^/RJ models. In conclusion, our data suggest that the nonintegrating method using mRNA has potential for cell therapy.

## 1. Introduction

Liver disease is a serious public health issue worldwide because of its high prevalence and poor long-term prognosis including cirrhosis, hepatocellular carcinoma, and premature death from liver failure [1, 2]. Furthermore, injuries with acquired, traumatic, or genetic etiologies can prevent the liver from performing a number of functions such as storing, detoxifying, and producing bile fluid and clotting factors and metabolic activities resulting in end-stage liver disease which ultimately requires liver transplantation [3–5]. Therefore, generating large quantities of hepatocytes is of paramount importance for scientists and clinicians. The ability of stem cells to be used in cell therapy has enormous potential [6]. Pluripotent stem cells have been used to generate hepatocyte-like cells [7–10]. Despite the usefulness of pluripotent stem cells, the risk of tumor formation [11, 12], long-term differentiation failure [13], and low differentiation efficiency [14] have emerged as points of controversy. The direct conversion of fibroblasts into target cells became feasible through lineage-specific transcription factors (TFs), and the direct conversion process is simpler and faster than induced pluripotent stem cell (iPSC) generation [15, 16]. Direct conversion of one cell type into another without using a pluripotent intermediate is a promising practical source for invaluable cells such as hepatocytes [17].

Compared to pluripotent stem cell differentiation, direct reprogramming has a number of advantages, including the lack of tumorigenic risk [18], a fast conversion rate [19], and the promise of injured tissue repair using* in vivo* reprogramming [20, 21]. Recently, a number of studies have investigated the results of direct conversion by RNA in cells such as neurons and cardiomyocyte-like cells [22, 23]; however, insufficient studies have been carried out in hepatocytes. We propose a method of functional hepatocyte generation suitable for engrafting in a damaged liver animal model, in which modified mRNA is used to overexpress reprogramming factors without genomic modification.

## 2. Materials and Methods

### 2.1. mRNA Synthesis by In Vitro Transcription (IVT)

To make mRNAs, template DNAs were obtained from Foxa3 and HNF4*α* plasmid. mRNAs were transcribed in vitro from 1.5 ug of each DNA template and synthesized using the MEGAscript T7 kit (Ambion, Austin, TX, USA), per each 40 ul of reaction buffer. IVT reactions were mixed with 2 ul of each NTP and incubated between 2 and 4 hrs at 37°C. To remove the template DNAs, 1ul of TURBO DNase was used after IVT reaction and incubated for 15 min at 37°C and purified with 70% EtOH for 5 min. Reacted mRNAs were capped during m^7^G capping and 2′-O-Methylation (ScriptCap m^7^G capping system and 2′-O-Methyltransferase kit, CELLSCRIPT, Madison, WI, USA), subsequently tailed (A-Plus Poly (A) Polymerase Tailing kit; CELLSCRIPT), and repurified as previously described. mRNA length was confirmed using 1% LE Agarose Gels (GenomicsOne Co. Ltd., Seoul, Korea). RNA concentrations were calculated with the use of Nanodrop and were adjusted to 200-300 ng/ul by adding Nuclease-free water (Ambion). As a control, eGFP mRNA was used and the expression of eGFP was observed and compared with Foxa3 and HNF4*α*.

### 2.2. Modified mRNA Transfection

To generate R-iHeps, mouse embryonic fibroblasts (MEFs) were cultured in Dulbecco's Modified Eagle's Medium (Life Technologies, Carlsbad, CA, USA) supplemented with 10% fetal bovine serum, 3.14 uM *β*-mercaptoethanol (Sigma-Aldrich, St. Louis, MO, USA), and 1% penicillin/streptomycin (Life Technologies) at 37°C in a CO_2_ incubator. Lipofectamine 2000 (Life Technologies) was used for mRNA transfection.  On day 0 and 3, 1.5 ug of Foxa3 and HNF4*α* mRNA each and 3 ul of lipofectamine 2000 were diluted in a mixture of 125 ul of Opti-MEM reduced serum media (Life Technologies) in separate tubes. They were then mixed together into one tube and were incubated at room temperature for 5 minutes. In a culture dish, 250 ul of the incubated mixture was added in 1ml of cell growth media and was incubated at 37°C for 4 hours. After 24 hours, the medium was changed with DMEM/F-12 (Life Technologies) supplemented with 10% fetal bovine serum (Life Technologies), 10mM Nicotinamide (Sigma-Aldrich), 0.1 uM dexamethasone (Sigma-Aldrich), 1% Insulin-Transferrin-Selenium-X Supplement (Life Technologies), 1% penicillin/streptomycin (Life Technologies), 20 ng/ml hepatocyte growth factor (Peprotech, Rocky Hill, NJ, USA), and 20 ng/ml epidermal growth factor (Peprotech). The medium was changed every two days.

### 2.3. Quantitative Real-Time PCR

One ug of mRNA isolated with Trizol reagent (Life Technologies) was reverse transcribed with the Transcriptor First Strand cDNA Synthesis Kit (Roche, Basel, Switzerland). Then, quantitative real-time PCR was performed using 10 ul of qPCR PreMix (Dyne Bio, Seongnam-si, Gyeonggi-do, Korea), 1 ul cDNA, and oligonucleotide primers on a CFX Connect Real-Time PCR Detection System (Bio-Rad, Hercules, CA, USA). Reactions were analyzed in triplicate for each gene. A total of 40 PCR cycles were performed, each cycle at 95°C for 20 sec, then 60°C for 40 sec. Melting curves and melting peak data were obtained to characterize PCR products. All primers are shown in Supplementary Table 1.

### 2.4. Immunostaining

The cells were fixed in 4% paraformaldehyde in phosphate buffered saline (PBS, pH 7.4) for 20 min at room temperature. The fixed cells were washed twice with a staining solution of PBS containing 1% fetal bovine serum for 5 min and then permeabilized with 0.25% Triton X-100 for 30 min at room temperature. Thereafter, the cells were incubated overnight at 4°C with the following primary antibodies: anti-albumin, E-cadherin, CK18, HNF4a, CYP1A2, ASGR1, Hep par-1, AFP, and vimentin (Table S2). The next day, cells were washed three times with a staining solution, and the appropriate fluorescence labeled Alexa-Fluor secondary antibody was added and incubated for 2 hours, in the dark, at room temperature. The nucleus was counterstained with Hoechst 33342 (Invitrogen, Carlsbad, CA, United States).

### 2.5. ICG Uptake and PAS Staining

For the indocyanine green (ICG) uptake assay, the cells were incubated for 15 min at room temperature with 1mg/ml DID Indocyanine Green Inj. (Dongindang Pharmaceutical, Siheung-si, Gyeonggi-do, Korea) and washed three times with PBS. For periodic acid-Schiff (PAS) staining, Periodic Acid-Schiff staining kit (Abcam, Cambridge, UK) was used. First, the cells were fixed with 4% paraformaldehyde in PBS for 20 min at room temperature. These fixed cells were rinsed in slow running tap water and then exposed to periodic acid solution for 5 min at room temperature. After being washed four times with distilled water, the cells were treated with Schiff's reagent for 15 min at room temperature and washed three times with distilled water. Thereafter, the cells were stained with hematoxylin (Modified Mayer's) for 2 min and washed three times with distilled water. A bluing reagent was applied for 30 sec to clearly identify the stained cells.

### 2.6. Albumin Secretion

To assess the function of these R-iHeps, we measured the secretion of the most well-known hepatic marker, albumin. Albumin secretion in R-iHeps was done according to the manufacturer's protocol using the Mouse Albumin ELISA kit (Bethyl Laboratories, Montgomery, TX, USA). Media was collected every two days and were stored at -80°C. The undiluted samples were measured in duplicate following the protocol's suggestion.

### 2.7. In Vivo Experiment

To determine whether R-iHeps can engraft and differentiate into functional hepatocytes in vivo, we used a liver injury mouse model, Alb-TRECK/SCID (kind gift from Taniguchi Hideki, Yokohama City University, Japan) and Fah^1R^Tyr^c^/RJ (kind gift from Hyongbum (Henry) Kim, Yonsei University). The animal experiments were performed in accordance with the Center for Laboratory Animal Sciences, the Medical Research Coordinating Center, and the HYU Industry-University Coordinating Foundation regulations (2016-0212A, 2017-0055A). To induce liver injury, Alb-TRECK/SCID mice were intraperitoneally injected with 2 ug/kg of diphtheria toxin (Sigma-Aldrich) 2 days before transplantation. Liver damage was also induced in Fah^1R^Tyr^c^/RJ mice by withdrawing NTBC ((2-(2-nitro-4-trifluoromethylbenzoyl)-1,3-cyclohexanedione)) 24 hrs before transplantation. mCherry-positive R-iHeps were obtained via FACS sorting and were transplanted through the spleen of the mouse (5x10^5^ cells/mice). Alb-TRECK/SCID and Fah^1R^Tyr^c^/RJ mice were sacrificed at 48 hrs and three weeks after transplantation, respectively.

## 3. Results

### 3.1. In Vitro Transcription and Expression of mRNA

**Figure 1:**
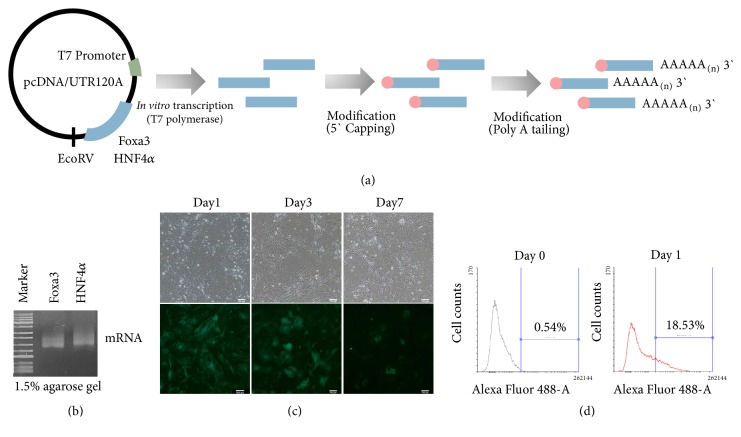
Transcription and expression of modified mRNA of HNF4*α* and Foxa3. (a) Scheme of in vitro transcription and modification of mRNA. (b) Gel loading of HNF4*α* and Foxa3 mRNA. (c) One time transfection and protein expression of green fluorescence protein mRNA into MEFs for 7 days. Green fluorescence was detected under fluorescence microscope. Scale bars: 100 um. (d) Analysis of transfection efficiency of GFP mRNA by Flow Cytometry.

To synthesize mRNA of Foxa3 and HNF4*α*, we cloned cDNA into pcDNA/UTR120A (Figure 1(a)). We conducted in vitro transcription using T7 polymerase and then modified synthesized mRNA. Synthesized mRNA is loaded in 1.5% agarose gels to confirm mRNA degradation. Foxa3 and HNF4*α* mRNA are synthesized to full length and not degraded (Figure 1(b)). mRNA stability and expression are evaluated for GFP mRNA transfection into MEFs (Figure 1(c)). After GFP mRNA transfection, GFP fluorescence was detected on day 1 and 3 under fluorescence microscope. However it almost disappeared on day 7.  The transfection efficiency of GFP mRNA was 18.53% onday 1 (Figure 1(d)). Therefore we decided transfection time of Foxa3 and HNF4*α* mRNA on day 0 and 3 to convert them into hepatocyte-like cells.

### 3.2. Generation of R-iHeps from MEFs and Morphogenesis of Hepatocyte-Like Cells

**Figure 2:**
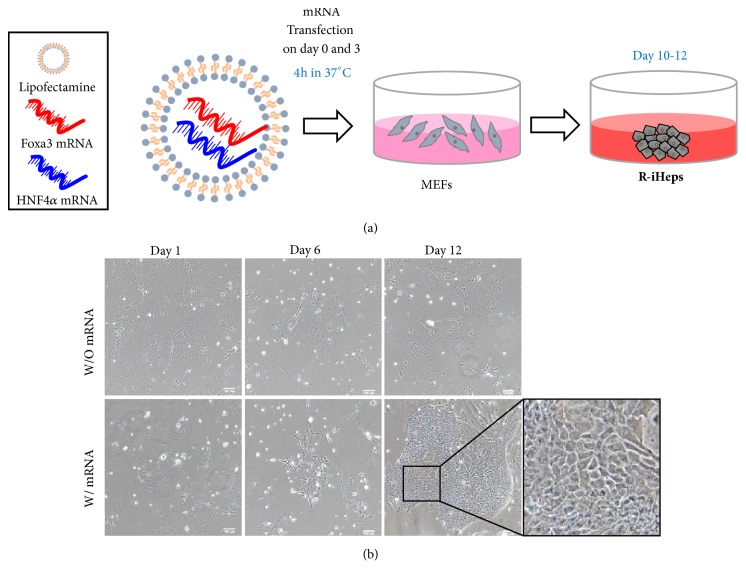
Generation of R-iHeps using mRNA from MEFs. (a) Scheme of generation of R-iHeps. mRNAs of modified HNF4*α* and Foxa3 were transfected with lipofectamine on day 0 and 3. MEFs: mouse embryonic fibroblasts; R-iHeps: RNA induced hepatocyte-like cells. (b) The morphology of directly converted R-iHeps by mRNA. On day 12, R-iHeps were shown and grown. Insets: higher magnification of the boxed areas. Scale bars: 100 um.

In order to generate hepatocyte-like cells, Foxa3 and HNF4*α* mRNA were transfected into mouse embryonic fibroblasts (MEFs) for 4 hours at temperature of 37°C (Figure 2(a)) on day 0 and 3. Two days after transfection, we switched media to direct conversion media for effective conversion into hepatic lineage. On day 6, MEFs started moving and switching morphology steadily (Figure 2(b)). Finally we found epithelial colonies similar with hepatocyte which are plentiful cytosol, small nuclei, and forming bile canaliculi after 12 days after transfection. These results suggest that directly converted R-iHeps are effective for generating hepatocyte-like cells from MEFs using mRNA.

### 3.3. Acquisition of Hepatic Characteristics of R-iHeps

**Figure 3:**
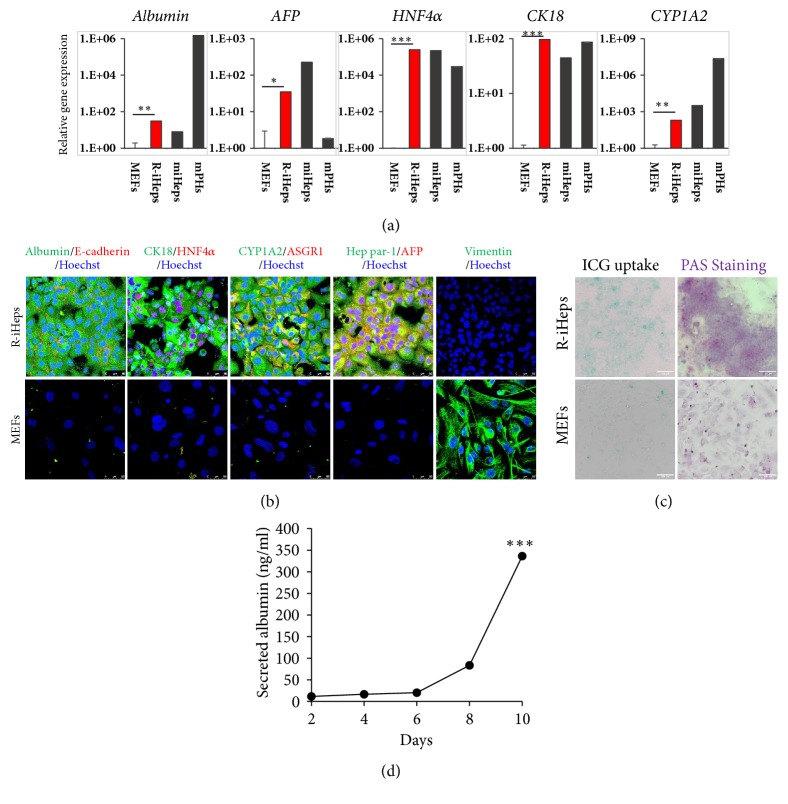
Analysis of hepatic characteristics of R-iHeps. (a, b) Comparison of hepatic gene and protein marker expression of R-iHeps and MEFs. (a) Expression levels of hepatic marker genes in R-iHeps (red bar) as determined by qPCR. Albumin, AFP, HNF4*α*, CK18, and CYP1A2 expression were increased in R-iHeps. MEFs: mouse embryonic fibroblasts; R-iHeps: RNA induced hepatocyte-like cells; miHeps: directly converted hepatocyte-like cells using retrovirus; mPHs: mouse primary hepatocytes. *∗*,* p*<.05; *∗∗*,* p*<.01; *∗∗∗*,* p*<.001. (b) Albumin (green)/E-cadherin (red), CK18 (green)/HNF4*α* (red), CYP1A2 (green)/ASGR1 (red), and Hep par-1 (green)/AFP (red) protein expression were detected in R-iHeps. Vimentin which is a fibroblast marker was detected not in R-iHeps but MEFs. Hoechst (blue) labels all nuclei. The images were captured using confocal microscopy. Scale bars: 50 um. (c) Confirmation of hepatic transporter function and presence of glycoprotein in R-iHeps by indocyanine green (ICG) uptake and Periodic Acid-Schiff (PAS) staining, respectively. (d) Measurement of secreted albumin in the culture media in vitro by ELISA. *∗∗∗*,* p*<.001.

To gain a better understanding of R-iHeps characteristics, we performed quantitative real-time PCR (qPCR) of hepatocyte-specific genes. Albumin, alpha-fetoprotein (AFP), HNF4*α*, CK18, and CYP1A2 expressions were markedly increased in R-iHeps as compared to MEFs (Figure 3(a)). Also, these genes' expressions were similar to miHeps which were generated using Foxa3 and HNF4*α* retrovirus [24, 25] and were correlated with protein expression (Figure 3(b)). Albumin, E-cadherin, CK18, HNF4*α*, CYP1A2, ASGR1, Hep par-1, and AFP were expressed in R-iHeps but not MEFs. Vimentin which is a fibroblast marker was only stained in MEFs. To evaluate hepatic function of R-iHeps* in vitro*, glycogen storage was revealed through Periodic Acid-Schiff (PAS) staining by more than 70% of the glycogen storage in R-iHeps and increased uptake of Indocyanine green (ICG) uptake compared to MEFs. This proved the xenobiotic metabolic activities in more than 50% of the R-iHeps which showed effective hepatic function (Figure 3(c)). In addition, the albumin secretion rate of R-iHeps was measured by Enzyme-Linked Immunosorbent Assay (ELISA) in the culture media (Figure 3(d)). Albumin secretion of R-iHeps (1x10^5^ cells) rapidly increased six days after seeding. This indicates that R-iHeps secrete albumin abundantly after stabilization period. These findings demonstrate that R-iHeps generated by the mRNA of Foxa3 and HNF4*α* could be another cell source of hepatocyte-like cells representing hepatic marker gene, protein expression, and a gain of hepatic function.

### 3.4. In Vivo Transplantation of R-iHeps

**Figure 4:**
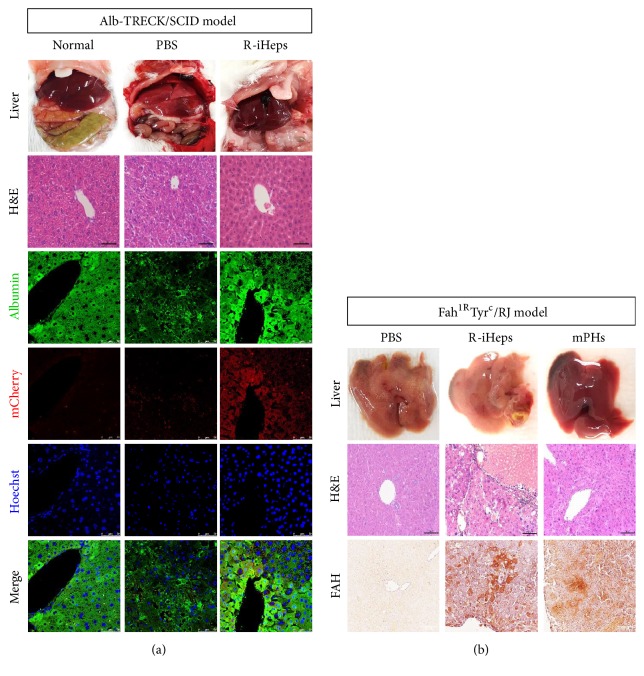
In vivo transplantation of R-iHeps. (a) mCherry labeled R-iHeps (5X10^5^ cells/100 ul) transplanted into Alb-TRECK/SCID mice via intrasplenic injection. Alb-TRECK/SCID mice were liver damaged by diphtheria toxin (DT, 2 ug/kg) 48 hrs before cell transplantation. All histological data were shown at 48 hrs after cell transplantation. Normal group: no DT administered; PBS group: PBS injection only after DT injury; R-iHeps group: R-iHeps injection after DT injury. Hoechst 33342 (blue) labels all nuclei. Scale bars in H&E staining picture: 100 um; scale bars in fluorescence pictures: 50 um. (b) R-iHeps (5X10^5^ cells/100 ul) transplanted into Fah^1R^Tyr^c^/RJ mice via intrasplenic injection. Fah^1R^Tyr^c^/RJ mice were liver damaged by NTBC ((2-(2-nitro-4-trifluoromethylbenzoyl)-1,3-cyclohexanedione)) withdrawal 24 hrs before cell injection. PBS group: PBS injection only; R-iHeps group: R-iHeps injection; mPHs group: mouse primary hepatocytes (5X10^5^ cells/100 ul) injection. Detection of FAH enzyme expression by immunoperoxidase staining at 3 weeks after transplantation. Scale bars: 100 um.

Finally, we implanted R-iHeps into two fulminant hepatic failure models to test whether engraftment and differentiation into functional hepatocytes in damaged liver could occur. First, we used Alb-TRECK/SCID model mice which were injured by diphtheria toxin (DT) [26]. mCherry tagged R-iHeps (5X10^5^ cells/mice), labeled for easy tracing in vivo, were administrated into the spleen 48 hrs after DT injection (2 ug/kg). At two days after transplantation, livers were harvested and sectioned. Histologically damaged liver (PBS injection group) showed disrupted cell junctions, necrotic cells were also found in H&E staining, and albumin expression was significantly decreased as seen through many unstained hepatocytes observed under confocal microscopy as compared to normal and R-iHeps injection groups (Figure 4(a)). On the other hand, in R-iHeps injection group, albumin positive hepatocytes costained with mCherry were found around blood vessels. In addition, liver structure was recovered by R-iHeps injection as shown in H&E staining. To prove the above data, R-iHeps were transplanted into Fah^1R^Tyr^c^/RJ mice model which was damaged by the withdrawal of NTBC ((2-(2-nitro-4-trifluoromethylbenzoyl)-1,3-cyclohexanedione)) [27]. Being transplanted after three weeks, R-iHeps and mouse primary hepatocytes (mPHs) were detected through fumarylacetoacetate hydrolase (FAH) enzyme (Figure 4(b)). Fah^1R^Tyr^c^/RJ mice model did not express FAH, but R-iHeps or mPHs transplanted mice liver produced FAH enzyme. Taken together, these results suggest that mRNA induced hepatocyte-like cells (R-iHeps) not only are transplantable in fulminant damaged liver, but also express the hepatic specific enzyme in vivo. Therefore, R-iHeps might be another cell source for liver regeneration.

## 4. Discussion

Patients with end-stage chronic liver disease generally require liver transplantation as the sole definitive method of treatment [28, 29]. Potential liver transplant recipients are outstripping possible donors [30]. Numerous studies have investigated ways to surmount this shortage [3]. The introduction of lineage-specific TFs into somatic cells enabled distinct cellular identities to be introduced, while bypassing a pluripotent stem cell state [31–34]. However, viral transduction systems have the potential risk of insertional mutations and integration-associated genotoxicity [35–38]. We propose a simple method of forming hepatocyte-like cells without relying on retroviral vectors. Our method successfully induced direct reprogramming of mouse embryonic fibroblasts into R-iHeps by mRNA transfection. Our data proved that R-iHeps, functionally similar to hepatocytes, were produced through direct reprogramming with mRNA. The R-iHeps showed a markedly increased expression of albumin and AFP, which are widely known as hepatocyte-specific proteins, while the expression of fibroblast-specific proteins such as vimentin decreased. In addition, PAS staining showed an increase in glycogen storage capacity, and ICG uptake confirmed that the cells effectively performed hepatic functions. Increases in albumin secretion and urea synthesis were confirmed by ELISA.

## 5. Conclusion

This study showed that mRNA can be utilized for direct hepatocyte reprogramming and that this technique is beneficial because it allows accurate control of reprogramming factors. As it has a number of advantages over traditional methods using retroviral vectors, our model has revealed a new paradigm with exciting potential for cell therapy with clinical applications.

## Data Availability

The data used to support the findings of this study are available from the corresponding author upon request.

## Supplementary Materials

Supplementary Materials Supplementary Table 1. Oligonucleotide primers that were used for quantitative real-time PCR. Supplementary Table 2. Antibodies that were used for immunofluorescence staining.

## References

[1] Wang F.-S., Fan J.-G., Zhang Z., Gao B., Wang H.-Y. (2015). The global burden of liver disease: the major impact of China. *Hepatology*.

[2] Nguyen V. T. T., Law M. G., Dore G. J. (2009). Hepatitis B-related hepatocellular carcinoma: Epidemiological characteristics and disease burden. *Journal of Viral Hepatitis*.

[3] Kwon Y. J. I., Lee K. G. E., Choi D. (2015). Clinical implications of advances in liver regeneration. *Clinical and Molecular Hepatology*.

[4] Rehm J., Samokhvalov A. V., Shield K. D. (2013). Global burden of alcoholic liver diseases. *Journal of Hepatology*.

[5] Lazo M., Hernaez R., Bonekamp S. (2011). Non-alcoholic fatty liver disease and mortality among US adults: prospective cohort study. *BMJ*.

[6] Jang Y. O., Jun B. G., Baik S. K., Kim M. Y., Kwon S. O. (2015). Inhibition of hepatic stellate cells by bone marrow-derived mesenchymal stem cells in hepatic fibrosis. *Clinical and Molecular Hepatology*.

[7] Schwartz R. E., Fleming H. E., Khetani S. R., Bhatia S. N. (2014). Pluripotent stem cell-derived hepatocyte-like cells. *Biotechnology Advances*.

[8] Nagamoto Y., Takayama K., Ohashi K. (2016). Transplantation of a human iPSC-derived hepatocyte sheet increases survival in mice with acute liver failure. *Journal of Hepatology*.

[9] Sullivan G. J., Hay D. C., Park I.-H. (2010). Generation of functional human hepatic endoderm from human induced pluripotent stem cells. *Hepatology*.

[10] Yi F., Liu G.-H., Belmonte J. C. I. (2012). Human induced pluripotent stem cells derived hepatocytes: Rising promise for disease modeling, drug development and cell therapy. *Protein & Cell*.

[11] Lee A. S., Tang C., Rao M. S., Weissman I. L., Wu J. C. (2013). Tumorigenicity as a clinical hurdle for pluripotent stem cell therapies. *Nature Medicine*.

[12] Miura K., Okada Y., Aoi T. (2009). Variation in the safety of induced pluripotent stem cell lines. *Nature Biotechnology*.

[13] Hannoun Z., Steichen C., Dianat N., Weber A., Dubart-Kupperschmitt A. (2016). The potential of induced pluripotent stem cell derived hepatocytes. *Journal of Hepatology*.

[14] Song Z., Cai J., Liu Y. (2009). Efficient generation of hepatocyte-like cells from human induced pluripotent stem cells. *Cell Research*.

[15] Du Y., Wang J., Jia J. (2014). Human hepatocytes with drug metabolic function induced from fibroblasts by lineage reprogramming. *Cell Stem Cell*.

[16] Zhu S., Rezvani M., Harbell J. (2014). Mouse liver repopulation with hepatocytes generated from human fibroblasts. *Nature*.

[17] Simeonov K. P., Uppal H. (2014). Direct reprogramming of human fibroblasts to hepatocyte-like cells by synthetic modified mRNAs. *PLoS ONE*.

[18] Ben-David U., Benvenisty N. (2011). The tumorigenicity of human embryonic and induced pluripotent stem cells. *Nature Reviews Cancer*.

[19] Vierbuchen T., Wernig M. (2011). Direct lineage conversions: Unnatural but useful?. *Nature Biotechnology*.

[20] Qian L., Huang Y., Spencer C. I. (2012). In vivo reprogramming of murine cardiac fibroblasts into induced cardiomyocytes. *Nature*.

[21] Song K., Nam Y. J., Luo X. (2012). Heart repair by reprogramming non-myocytes with cardiac transcription factors. *Nature*.

[22] Lee K., Yu P. Z., Lingampalli N., Kim Y. J., Tang R., Murthy N. (2015). Peptide-enhanced mRNA transfection in cultured mouse cardiac fibroblasts and direct reprogramming towards cardiomyocyte-like cells. *International Journal of Nanomedicine*.

[23] Xue Y., Ouyang K., Huang J. (2013). Direct conversion of fibroblasts to neurons by reprogramming PTB-regulated MicroRNA circuits. *Cell*.

[24] Kang K., Kim Y., Jeon H. (2018). Three-Dimensional Bioprinting of Hepatic Structures with Directly Converted Hepatocyte-Like Cells. *Tissue Engineering Part: A*.

[25] Cho Y.-D., Yoon S., Kang K. (2017). Simple Maturation of Direct-Converted Hepatocytes Derived from Fibroblasts. *Tissue Engineering and Regenerative Medicine*.

[26] Zhang R.-R., Zheng Y.-W., Li B. (2015). Human hepatic stem cells transplanted into a fulminant hepatic failure Alb-TRECK/SCID mouse model exhibit liver reconstitution and drug metabolism capabilities. *Stem Cell Research & Therapy*.

[27] Holme E., Lindstedt S. (1998). Tyrosinaemia type I and NTBC (2-(2-nitro-4-trifluoromethylbenzoyl)-1,3- cyclohexanedione). *Journal of Inherited Metabolic Disease*.

[28] Lee H. W., Suh K.-S. (2016). Liver transplantation for advanced hepatocellular carcinoma. *Clinical and Molecular Hepatology*.

[29] Florman S., Miller C. M. (2006). Live donor liver transplantation. *Liver Transplantation*.

[30] Dutkowski P., Oberkofler C. E., Béchir M. (2011). The model for end-stage liver disease allocation system for liver transplantation saves lives, but increases morbidity and cost: A prospective outcome analysis. *Liver Transplantation*.

[31] Vierbuchen T., Ostermeier A., Pang Z. P., Kokubu Y., Südhof T. C., Wernig M. (2010). Direct conversion of fibroblasts to functional neurons by defined factors. *Nature*.

[32] Pang Z. P., Yang N., Vierbuchen T. (2011). Induction of human neuronal cells by defined transcription factors. *Nature*.

[33] Sekiya S., Suzuki A. (2011). Direct conversion of mouse fibroblasts to hepatocyte-like cells by defined factors. *Nature*.

[34] Huang P., Zhang L., Gao Y. (2014). Direct reprogramming of human fibroblasts to functional and expandable hepatocytes. *Cell Stem Cell*.

[35] Vakas F., Stadtfeld M., De Andres-Aguayo L. (2009). Fibroblast-derived induced pluripotent stem cells show no common retroviral vector insertions. *Stem Cells*.

[36] Winkler T., Cantilena A., Métais J.-Y. (2010). No evidence for clonal selection due to lentiviral integration sites in human induced pluripotent stem cells. *Stem Cells*.

[37] Wu C., Dunbar C. E. (2011). Stem cell gene therapy: The risks of insertional mutagenesis and approaches to minimize genotoxicity. *Frontiers of Medicine in China*.

[38] Hong S. G., Dunbar C. E., Winkler T. (2013). Assessing the risks of genotoxicity in the therapeutic development of induced pluripotent stem cells. *Molecular Therapy*.

