# Supplementary material for: Nonintegrating Direct Conversion Using mRNA into Hepatocyte-Like Cells
Source: Biomed Res Int. 2018 Sep 20;2018:8240567. doi: 10.1155/2018/8240567 (PMC6171260; doi:10.1155/2018/8240567)
Supplement: Supplementary Materials — Supplementary Table 1. Oligonucleotide primers that were used for quantitative real-time PCR. Supplementary Table 2. Antibodies that were used for immunofluorescence staining. [file 8240567.f1.docx]

**Supplementary Materials**

**Supplementary Table 1.** Oligonucleotide primers that were used for quantitative Real-Time PCR

| **Gene** | **Primer** | **Primer Sequence (5’ –> 3’)** |
| --- | --- | --- |
| **Albumin** | F | GGCTACAGCGGAGCAACTGA |
|  | R | GCCTGAGAAGGTTGTGGTTGTG |
| **AFP** | F | AGCCTGAACTGACAGAGGAGCA |
|  | R | TAAACGCCCAAAGCATCACG |
| **HNF4a** | F | ATCGTCAAGCCTCCCTCTGC |
|  | R | GACTGGTCCCTCGTGTCACATC |
| **CK18** | F | GACTGGGGCCACTACTTCAA |
|  | R | CATCTACCACCTTGCGGAGT |
| **CYP1a2** | F | AGGAGCTGGACACGGTGGTT |
|  | R | AGGTGTCCCTCGTTGTGCTG |
| **GAPDH** | F | CCAATGTGTCCGTCGTGGAT |
|  | R | TTGCTGTTGAAGTCGCAGGAG |

**Supplementary Table 2**. Antibodies that were used for immunofluorescence staining

| **Antibodies** | **Products Number (Company)** |
| --- | --- |
| **Albumin** | ab19194 (Abcam) |
| **E-Cadherin** | ab76055 (Abcam) |
| **CYP1A2** | ab22717 (Abcam) |
| **ASGR1** | ab49355 (Abcam) |
| **Hep par-1** | 264M-9 (Sigma Aldrich) |
| **AFP** | A0008 (DAKO) |
| **Vimentin** | MA5-14564 (Thermo fisher) |
